# Matched-pair comparisons of minimally invasive esophagectomy versus open esophagectomy for resectable esophageal cancer: A systematic review and meta-analysis protocol

**DOI:** 10.1097/MD.0000000000011447

**Published:** 2018-07-13

**Authors:** Wei Wang, Feiyu Liu, Tao Hu, Chaoyang Wang

**Affiliations:** a Department of Thoracic Surgery, The Affiliated Yantai Yuhuangding Hospital of Qingdao University, Yantai, China; b Department of Thoracic and Cardiovascular Surgery, The University of Texas MD Anderson Cancer Center, Houston, TX, USA; c Department of Pharmacy, The Affiliated Yantai Yuhuangding Hospital of Qingdao University, Yantai, China.

**Keywords:** esophageal neoplasms, esophagectomy, minimally invasive surgical procedures

## Abstract

**Background:**

Open esophagectomy (OE) with radical lymphadenectomy is known as one of the most invasive digestive surgeries with the high rate of complications. Minimally invasive esophagectomy (MIE) has developed very rapidly and has formed several available technical approaches. This systematic review and meta-analysis is aiming at how beneficial, and to what extent MIE resection really will be.

**Methods:**

A systematic literature search will be performed through May 31, 2018 using MEDLINE, EMBASE, the Cochrane Central Register of Controlled Trials, and Google Scholar for relevant articles published in any language. Randomized controlled trials, prospective cohort studies, and propensity score matched comparative studies will be included. If data are sufficient, subgroup analyses will be conducted in different surgical procedures of MIE.

**Results:**

The results of this systematic review and meta-analysis will be published in a peer-reviewed journal.

**Conclusion:**

This will be the first systematic review and meta-analysis using data of randomized controlled, prospective, and propensity score matched comparative studies to compare the outcomes between MIE and OE updating to May 31, 2018.

## 1 Introduction

Esophageal cancer now is the ninth most commonly diagnosed cancer and the sixth most common cause of cancer-related deaths worldwide in 2013.^[1]^ And it is one of the worst malignant digestive neoplasms with poor treatment outcomes. Esophagectomy is the mainstay of curative treatment strategies for localized esophageal cancer,^[2–5]^ which plays an important role and offers a potential curable chance for these patients.

However, the type of surgical procedures and the extent of lymphadenectomy which are necessary for esophageal cancer patients remain controversial.^[6,7]^ Traditionally, surgical treatment means open esophagectomy (OE) which is associated with high morbidity and mortality via transthoracic or transhiatal approaches. Respiratory complications are very common with OE, which can increase the risk of death up to about 20%.^[8,9]^ So esophagectomy with radical lymphadenectomy is known as one of the most invasive digestive surgeries. Therefore, Esophagectomy using thoracoscopic and/or laparoscopic approaches as a less invasive surgery seems to be very attractive. In 1992, Cuschieri et al^[10]^ first reported performing minimally invasive esophagectomy (MIE) via thoracoscopy. Since then, many institutions have described various modalities for MIE.^[11–15]^ It has developed very rapidly and has formed several available technical approaches utilizing combinations of standard thoracoscopy, laparoscopy, and more recently, robotic assistance.

Several studies have shown MIE has an advantage in perioperative complications and quality of life, with seemingly comparable oncologic outcomes.^[15–23]^ But these available evidences are very heterogeneous because most of the studies were retrospective and case-control studies of low quality with a high risk of bias. It is still unclear how beneficial, and to what extent MIE resection really is. And this is not conducive for MIE to spread.^[24]^ Due to some high quality studies were published recently, we can take this advantage to conduct a systematic review and meta-analysis with higher level of evidences.^[25–29]^ Moreover, in order to minimize the heterogeneity and bias, we will select randomized controlled trials (RCTs) and propensity score matched comparative studies which matched across a range of baseline factors to generate 2 similar groups for comparison. If data are sufficient, we will also conduct subgroup analyses in different surgical procedures of MIE.

## 2 Objective

A systematic review and meta-analysis will be performed to assess the effects of MIE versus OE for patients with resectable esophageal cancer.

## 3 Methods

This protocol for systematic review and meta-analysis is performed according to the Preferred Reporting Items for Systematic Review and Meta-Analysis Protocols (PRISMA-P) statement.^[30]^ This protocol has been registered in the PROSPERO network (registration number: CRD42018085710). This systematic review and meta-analysis will be reported according to the Preferred Reporting Items for Systematic Reviews and Meta-Analyses (PRISMA) guidelines.^[31]^

### 3.1 Eligibility criteria

#### 3.1.1 Types of participants

The included participants will be adults who were diagnosed with esophageal cancer histologically or cytologically confirmed and treated with esophagectomy. There will be no restrictions regarding sex, race/ethnicity, education, and economic status.

#### 3.1.2 Types of studies

We propose to include studies that report comparisons between MEI and OE. RCTs, prospective cohort studies, and propensity score matched comparative studies will be used for the qualitative and quantitative synthesis of the systematic review. No language or date restrictions will be applied.

#### 3.1.3 Exclusion criteria

Non-peer reviewed articles, review articles, case reports, case series, animal studies, meeting abstracts, letters to the editor, commentaries, editorials, proceedings, nonpropensity-matched comparative studies and other nonrelevant studies will be excluded from analysis.

### 3.2 Information sources

We will perform a systematic literature search through May 31, 2018 using MEDLINE, EMBASE, the Cochrane Central Register of Controlled Trials, and Google Scholar for relevant articles published in any language.

### 3.3 Search strategy

The relevant searching terms will match Medical Subject Heading terms, and the searches will be repeated immediately before the final analyses to identify additional studies for inclusion. An example of the PubMed search strategy is shown in Table 1.

**Table 1:**
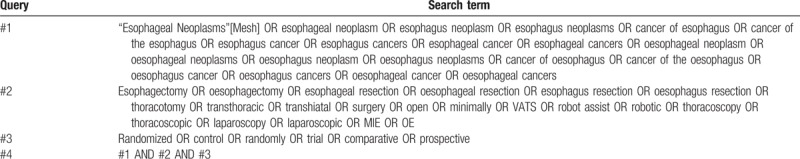
Search strategy for PubMed.

### 3.4 Study records

#### 3.4.1 Selection of studies

Two review authors (WW, FL) will independently screen titles and abstracts of all the potential studies to assess whether they meet the inclusion criteria as defined by the protocol. We will retrieve the full text of all potentially eligible studies and 2 review authors (WW, FL) will independently screen the full-text and identify studies for inclusion, and record reasons for exclusion of the ineligible studies. Any disagreement will be resolved through discussion or, if required, consultation with a third review author (TH or CW). Duplicates will be excluded and multiple reports of the same study will be integrated into one unit of interest in the review. The selection process will be recorded in sufficient detail to complete a PRISMA flow diagram and “Characteristics of excluded studies” table.^[32]^

#### 3.4.2 Data extraction and management

Data will be extracted from the included studies by 3 authors (WW, FL, TH) independently and recorded on a predesigned data collection form. We will extract the following study characteristics:

(1) *Study characteristics*: study design, number of study centers and locations, study setting, withdrawals, total duration of study, periods of data collection, follow-up duration, blanking periods.
(2) *Population characteristics*: number, mean age, age range, gender, diagnostic criteria, inclusion criteria, exclusion criteria, pathological confirmation, staging of the tumor according to the AJCC TNM classification for esophageal cancer.
(3) *Intervention characteristics*: surgical approach, duration, bleeding, transfusion, thoracotomy conversion.
(4) *Outcomes*: primary and secondary outcomes specified and collected, and time points reported.

### 3.5 Outcomes

#### 3.5.1 Primary outcomes

The primary outcomes are: overall survival (all-cause deaths will be included and measured from the date of participant randomization to the date of death or study end date if the participant was alive) and disease-free survival (DFS).

#### 3.5.2 Secondary outcomes

The secondary outcomes are blood loss, operative duration, number of lymph nodes retrieved, length of ICU stay, length of hospitalization, and complication rate. The complications are as follows: pulmonary complications, cardiovascular complications, gastrointestinal complications, surgical technology related complications (including anastomotic leak and reintervention).

### 3.6 Assessment of risk of bias

Two review authors (WW, FL) will independently assess the risk of bias for each study using the criteria outlined in the Cochrane Handbook for Systematic Reviews of Interventions. Any disagreements will be resolved by discussion or by involving another review author (CW or TH). The risk of bias will be assessed according to the following domains: Random sequence generation. Allocation concealment. Blinding of participants and personnel. Blinding of outcome assessment. Incomplete outcome data. Selective outcome reporting. Other bias. Each potential source of bias will be graded as high, low or unclear and a quote from the study report with a justification for our judgment will be provided in the “Risk of bias” table. The risk of bias judgments across different studies for each of the domains listed will be summarized.

### 3.7 Data synthesis

Data from studies judged to be clinically homogeneous will be pooled using Review Manager 5.3 software. Heterogeneity between studies will be assessed using the Cochran *Q* and Higgins *Ι*^2^ statistic. *P* < .10 for the Chi^2^ statistic or an *I*^2^ > 50% will be considered as showing considerable heterogeneity, and the data will be analyzed using the random-effect model. Otherwise, the fixed-effect model will be used. The Mantel–Haenszel method will be applied for pooling of dichotomous data and results will be presented as relative risk (RR) with their 95% confidence intervals (CIs). Inverse variance method will be used for pooling of continuous data and results will be presented as standardized mean difference (SMD) with their 95% CI.

#### 3.7.1 Subgroup analysis

If data are sufficient, we will conduct subgroup analyses in different surgical procedures of MIE. Subgroup analyses will also be performed to explore potential sources of heterogeneity.

#### 3.7.2 Sensitivity analysis

A sensitivity analysis will be performed to confirm whether the pooled results are robust and credible by excluding highly biased studies.

#### 3.7.3 Dealing with missing data

In the condition of missing or unclear data, study authors will be contacted at the eligibility assessment and/or data extraction stage. Secondary publications may be considered as missing data if they have the same study population.

### 3.8 Publication bias

Egger regression test will be performed to assess the publication bias of the included studies.^[33]^ If there is a publication bias, trim and fill analysis will be performed.

### 3.9 Evidence evaluation

The evidence grade will be determined by using the guidelines of the GRADE (Grading of Recommendations, Assessment, Development, and Evaluation) system using 4 levels—high quality, moderate quality, low quality, and very low quality.^[34]^

## 4 Discussion

Esophageal cancer is one of the most common digestive tract cancers worldwide. Esophagectomy plays an important role and offers a potential curable chance for these patients. But traditional OE is associated with high perioperative morbidity and mortality. There is growing evidence in literature that MIE may decrease morbidity. However, the type of surgical procedures and the extent of lymphadenectomy which are necessary for esophageal cancer patients remain controversial.

To our knowledge, this will be the first systematic review and meta-analysis using data of RCTs, prospective studies, and propensity-matched comparative studies to compare the clinical outcomes between MIE and OE updating to May 31, 2018. The aim of our study is to draw an objective conclusion of the comparisons on clinical outcomes and to provide physicians level I evidences for clinical decision makings. Each patient should be individually assessed to accept an appropriate therapeutic regimen based on the pathological type of cancer, location, local or regional involvement, and body functional status. Due to the difficulty and complexity of the procedures, there is still a long way for MIE to be popularized. We hope this fine surgical modality will contribute to human health.

## Author contributions

**Contributors:** Wei Wang, Feiyu Liu, and Chaoyang Wang conceived and designed this study. Wei Wang and Feiyu Liu drafted the protocol. Wei Wang, Feiyu Liu, and Tao Hu will conduct the search, data screening and extraction. Wei Wang, Feiyu Liu, Tao Hu, and Chaoyang Wang have critically reviewed the manuscript and approved it for publication.

**Conceptualization:** Chaoyang Wang.

**Data curation:** Wei Wang, Feiyu Liu.

**Formal analysis:** Wei Wang.

**Investigation:** Wei Wang.

**Methodology:** Wei Wang, Feiyu Liu, Tao Hu.

**Project administration:** Chaoyang Wang, Tao Hu.

**Resources:** Wei Wang, Chaoyang Wang, Feiyu Liu.

**Software:** Wei Wang, Feiyu Liu.

**Supervision:** Chaoyang Wang, Tao Hu.

**Validation:** Feiyu Liu.

**Visualization:** Chaoyang Wang, Tao Hu.

**Writing – original draft:** Wei Wang, Chaoyang Wang, Feiyu Liu.

**Writing – review & editing:** Wei Wang, Feiyu Liu.
